# Randomized Controlled Trials: A Systematic Review of Laparoscopic Surgery and Simulation-Based Training

**DOI:** 10.5539/gjhs.v7n2p310

**Published:** 2014-12-12

**Authors:** Allison A. Vanderbilt, Amelia C. Grover, Nicholas J. Pastis, Moshe Feldman, Deborah Diaz Granados, Lydia K. Murithi, Arch G. Mainous

**Affiliations:** 1 Center on Health Disparities, School of Medicine, Virginia Commonwealth University, Richmond, Virginia, USA; 2 School of Medicine, Virginia Commonwealth University, Richmond, Virginia, USA; 3 Division of Pulmonary and Critical Care, Medical University of South Carolina, Charleston, South Carolina, USA; 4 Office of Assessment and Evaluation Studies, School of Medicine, Virginia Commonwealth University, Richmond, Virginia, USA; 5 Department of Health Services Research, Management & Policy and Professor in the Department of Community Health and Family Medicine at the University of Florida, USA

**Keywords:** laparoscopic surgery, simulation, medical education, systematic review, skill transfer, translation, randomized clinical trials

## Abstract

**Introduction:**

This systematic review was conducted to analyze the impact and describe simulation-based training and the acquisition of laparoscopic surgery skills during medical school and residency programs.

**Methods:**

This systematic review focused on the published literature that used randomized controlled trials to examine the effectiveness of simulation-based training to develop laparoscopic surgery skills. Searching PubMed from the inception of the databases to May 1, 2014 and specific hand journal searches identified the studies. This current review of the literature addresses the question of whether laparoscopic simulation translates the acquisition of surgical skills to the operating room (OR).

**Results:**

This systematic review of simulation-based training and laparoscopic surgery found that specific skills could be translatable to the OR. Twenty-one studies reported learning outcomes measured in five behavioral categories: economy of movement (8 studies); suturing (3 studies); performance time (13 studies); error rates (7 studies), and global rating (7 studies).

**Conclusion:**

Simulation-based training can lead to demonstrable benefits of surgical skills in the OR environment. This review suggests that simulation-based training is an effective way to teach laparoscopic surgery skills, increase translation of laparoscopic surgery skills to the OR, and increase patient safety; however, more research should be conducted to determine if and how simulation can become apart of surgical curriculum.

## 1. Introduction

Laparoscopic surgery has become the “gold standard” for common surgical procedures such as cholecystectomies and appendectomies (Bennett, Birch, Menzes, Vizhul, & Karmali, 2011; Richardson, Carter, Fuhrman, Bolton, & Bowen, 2000), and is associated with less surgical trauma, faster postoperative recovery, shorter hospital stays, and better cosmetic results (Munz, Kumar, Moorthy, Bann, & Darzi, 2004; Johnson, & Walsh, 2009). There is a general understanding that simulation-based training improves knowledge (McGaghie, Siddall, Mazmanian, & Myers, 2009; Ehdaie, Tracy, Reynolds, Cung, Thomas, Floyd, & Schenkman, 2011) and that training outside the operating room (OR) reduces the risk of adverse surgical events (Hyltander, Liljegren, Rhodin, & Lönroth, 2002; Andreatta et al., 2006; Aggarwal, Ward, Balasundaram, Sains, Athanasiou, & Darzi, 2007).

As the health care community creates and maintains new teaching methods to train competent surgeons, learning opportunities that exist outside the OR are becoming a recommended method for developing laparoscopic surgery skills (Ahlberg et al., 2007; Jordan, Gallagher, McGuigan, McGlade, & McClure, 2000; Verdaasdonk, Dankelman, Lange, & Stassen, 2008). Training outside the OR reduces the risk of adverse surgical events (Hyltander, Liljegren, Rhodin, & Lönroth, 2002; Aggarwal, Ward, Balasundaram, Sains, Athanasiou, & Darzi, 2007; Ahlberg, Heikkinen, Iselius, Leijonmarck, Rutqvist, & Arvidsson, 2002). Simulation-based surgical skills and procedures allows inexperienced surgeons to acquire skills through repetitive practice in a safe, nonthreatening environment, prior to encountering the risk and time pressures inherent in the OR (Andreatta et al., 2006; Miskovic, Wyles, Ni, Darzi, & Hanna, 2010). Those responsible for designing simulation facilities work with limited evidence to resolve complex questions relating to education, translation of skills learned, and patient safety with regard to teaching laparoscopic surgery.

In a systematic review conducted in 2006, researchers found that learners acquire similar clinical results as surgeons in laparoscopic colorectal surgery, if supervised by an expert during training (Sutherland, Middleton, Anthony, Hamdorf, Cregan, Scott, & Maddern, 2006); however, this review was limited only to colorectal surgeries. In a different systematic review, investigators reported that simulation training may not be a better method than patients, cadavers, and animals for teaching surgical skills (Sutherland et al., 2006), but the skills learned by simulation-based training appeared to be transferable to the OR. This review conducted by Strum and researchers (Sturm, Windsor, Cosman, Cregan, Hewett, & Maddern, 2008) was limited to 11 published studies and was conducted in 2008. Gurusamy and colleagues (Gurusamy, Aggarwal, Palanivelu, & Davidson, 2008) found that virtual reality training can supplement laparoscopic surgery training, but variability across research designs and conflicting findings in the published studies prevented the confirmation of clear best practices. More recently, Cook and colleagues (Cook et al., 2001) studied technology-enhanced simulation training and concluded that simulation training is associated with large effects on clinician behaviors and moderate effects on patient care.

This current review of the literature addresses the question of whether laparoscopic simulation translates the acquisition of surgical skills to the OR. The conceptual framework for this manuscript is focused on the importance and relevance related to the education of surgical skills, the translation of surgical skills acquired outside of the OR, and improvements focused on safety for patients. A review of published research was completed to describe the impact of simulation-based training on the acquisition of laparoscopic surgery skills and the translation of these skills to the OR. Skills acquisition was assessed for performance time, global rating, suturing, cutting, and cautery skills; errors, and economy of movement.

## 2. Methods

**Figure 1:**
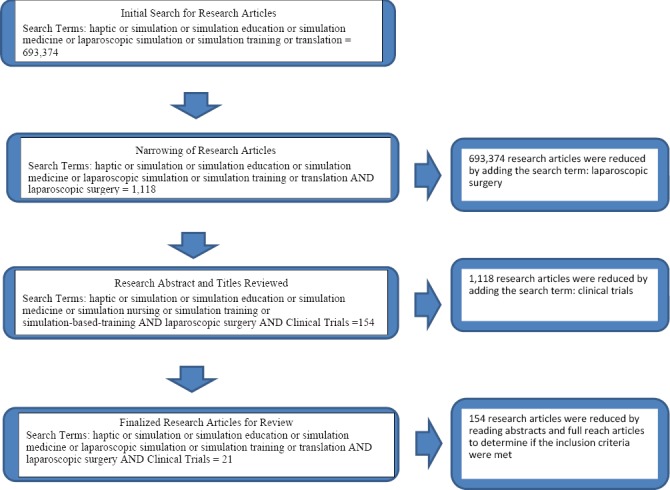
Flow chart for research article search in May 2014

This review focused on published literature that examines the effectiveness of simulation-based training to develop laparoscopic surgery skills translation into the OR. The studies reviewed were identified by searching PubMed from the inception of the database to April 1, 2014 and hand searching: Simulation in Health Care, Annals of Surgery, Journal American Surgery, International Journal of Surgery, Surgery, Archives of Surgery, and The British Journal of Surgery from 2000 – May 2014. Multiple combinations of several relevant key words were used to identify articles for review (haptic or simulation or simulation education or simulation medicine or laparoscopic simulation or simulation training or translation AND laparoscopic surgery). Figure 1 demonstrates the elimination of articles that came up during the search process.

### 2.1 Inclusion and Exclusion Criteria

Inclusion criteria required that studies: (a) use a randomized controlled design that includes at least one intervention group and one control group that either received no training or traditional training in the operating room, (b) single-group pretest-posttest, (c) two group nonrandomized, (d) parallel-group (e) crossover designs, (f) use simulation-based training as the educational intervention for teaching laparoscopic surgery skills, and (e) translation of skills was measured into the OR setting. Simulation-based training was defined broadly to include equipment that replicated the task environment with sufficient realism to serve as a training tool. Examples of the simulators included in this systematic review were box trainers, computer software, virtual reality simulators, task trainers, and high fidelity and static mannequins. The exclusion criteria were: (a) studies that did not use simulation as the educational intervention for teaching laparoscopic surgery skills, (b) literature reviews, and (c) translation of skills was not measured into the OR setting.

An adopted coding framework based on PRISMA (Liberati et al., 2001) and Cochrane handbook (Higgins, 2012) was used to review the literature. The first author independently coded each of the articles discovered through the literature search. When reviewing the literature some abstracts provided enough detail and information related to the methods to determine if the inclusion criteria were met; if not, the full manuscript was read to determine if the methods met the inclusion criteria. The manuscripts were eliminated because the methods did not meet the inclusion criteria.

## 3. Results

The results reported in this section are based on the 20 articles we determined met our inclusion criteria. Table 1 describes the types of simulators implemented in the 21 studies, manufacturers for the simulators, definitions for the simulators, and performance skills the simulators provide. A total of 21 studies were analyzed; the specific simulators, participants, assessments, and details of the 21 studies are provided in Tables 2 and 3. All post-training assessment were translational to either a porcine model or the OR, 9 (43%) studies conducted the posttest in a Porcine Model, 12 (57%) studies conducted the posttest in the OR with patients.

**Table 1:** Laparoscopic training tools, definitions, manufacturers, and procedures commonly trained in surgery

Type of Simulation	Definition	Manufacturer	Camera Navigation	Clipping & Cutting	Suturing & Knot Tying	Lifting & Grasping	Dissection
**Box Trainer**	A box that incorporates conventional laparoscopic equipment to perform basic skills, is versatile, and enables training on animal parts as well as synthetic inanimate models	Simulab Corporation	X	X	X	X	X
**Task Trainer**	A partial component of a simulator or simulation modality, for example, an arm, leg, or torso.	Limbs and Things		X	X		X
**MIST-VR**	A virtual reality simulator with six different tasks to simulate maneuvers performed during laparoscopic cholecystectomy in a computerized environment.	Mentice AB	X	X		X	X
**LapMentor/ LapMentor II**	A virtual reality simulator consisting of a camera and two calibrated working instruments for which the motion of the instruments is translated to a two-dimensional computer screen for student practices.	Simbionix Ltd.	X	X	X	X	X
**LapSim**	A computer-based simulator creating a virtual laparoscopic setting through a computer operating system, a video monitor, a laparoscopic interface containing two pistol-grip instruments, and a diathermy pedal without haptic feedback	Surgical Science	X	X	X	X	X
**EndoTower**	EndoTower software consists of an angled telescope simulator composed of rotating camera and telescopic components.	Verefi Technologies, INC.	X	X			
**MISTELS/ FLS trainer**	McGill Inanimate System for Training and Evaluation of Laparoscopic Skills – this inexpensive, portable, and flexible system allows students to practice in a virtual Endotrainer box.	SAGES		X	X	X	
**SIMENDO VR**	Computer software used to train eye-hand coordination skills by camera navigation and basic drills.	Delta Tech	X		X	X	
**URO Mentor**	A hybrid simulator, consisting of a personal computer based system linked to a mannequin with real endoscopes. Cytoscopic and ureterosciopic procedures are performed using either flexible or semi rigid endoscopes	Simbionix Ltd.	X	X		X	X
**Da Vinci Skills Simulator**	A portable simulator containing a variety of exercises and scenarios specifically designed to give users the opportunity to improve their proficiency with surgical controls.	Intuitive Surgical	X	X	X	X	X

**Table 2:** Study participants, pre-study data, simulation, features of training procedures, and assessment

Citation	Participants	Pre-study data collected	Simulation intervention	Additional training	Time between initial assessment and final assessment	Training time	Training tasks
**Aggarwal et al., 2007**	· 19 novice surgeons	· None	· LapSim VR	· None	· Not specified* · Final assessment conducted over 4 weeks	· Not specified*	· 7 basic tasks · 3 levels of difficulty · Skills for: 1) Instrument navigation 2) Grasping tissues 3) Clip application
**Ahlberg et al., 2007**	· 29 4^th^ year medical students	· None	· MIST-VR	· None	· Not specified*	· 3 hours	· 6 tasks simulate the maneuvers performed during a laparoscopic cholecystectomy.
**Ahlberg et al., 2002**	· 13 surgical residents: Unclear on study design*	· Mental rotation · Cognitive tests, · Verbal working memory · Attitude toward simulator	· LapSim	· None	· The 1^st^ surgery performed within 2 weeks of baseline measurement. · The last surgery performed within 6 months of the start.	· Maximum of 40 hours in 1 week	· Grasping · Lift grasp · Cutting right · Cutting left · Clip application
**Andreatta et al., 2006**	· 19 surgical interns 1) 10 in the training group 2) 9 in the control group	· Computer game experience	· Simbionix LapMentor	· None	· 4 weeks	· Duration is not specified* · At least 10 repetitions were performed in order to reach proficiency by trainees	· 30-degree camera navigation · Eye-hand coordination · Clipping and grasping · Cutting Electrocautery · Translocation of objects
**Banks et al., 2007**	· 20 postgraduate year 1 residents	· Laparoscopic experience	· Task Trainer · Laparoscopic BTL	· None	· 4 months	· Not specified, estimated to be approximately 4 hours*	· 1 hr. of didactics · 2 hours of hands on teaching in the skills lab with 3 stations: 1) Suturing pigs feet 2) Knot tying board 3) A lap simulator and an operative lap tower
**Bennet et al. 2011**	· 70 Medical Students	· Laparoscopic experience · Interest in surgical specialty · Comfort with angled laparoscope.	· Box Trainer	· None	· 6 weeks	· 10 minutes	· Tutorial on camera simulator navigation
**Gala et al., 2013**	· 44 residents (PGY 1 & 2) · 66 (PGY 3 & 4)	· Baseline data laparoscopic Pomeroy Bilateral tubal ligation	· Psychomotor board testing with a peg board test	· 2 times till mastery accomplished on all 5 validated laparoscopic simulators	· not reported	· 30 minutes with faculty member	· Clipping · Grasping · Lifting · Time · Peg transfer · Pattern cutting
**Ganai et al., 2007**	· 19 M3 students 1) 9 training group 2) 10 control group	· Laparoscopic cases observed or participated in were measured between baseline and performance	· Endo Tower	· None	· 3-4 weeks	· 1-hour sessions. · Limit of 10 sessions per difficulty level (3 levels). · Had to train to proficiency	· Navigation around a complex geometric structure to achieve specific view of target objects.
**Grantcharov et al., 2004**	· 16 surgical residents with limited laparoscopic experience	· None	· MIST-VR	· None	· 14 days	· 3 hours	· Task 1: virtual sphere to box transfer; · Task 2: hand to hand transfer · Task 3: grasping the segments of virtual pipe. · Task 4: grasp virtual sphere, touch tip of other instrument, withdraw and reinsert, and touch sphere again. · Task 5: virtual sphere was grasped, three plates appear on the surface of sphere, these are then touched by the other instruments. · Task 6: combines actions of 4 and 5 with diathermying the plates while holding the sphere
**Hogle et al., 2009**	· Study 1: 1) 6 trained 2) 6 control · Study 3: 3) 10 trained 4) 11 control	· Study 1and 3: None	· Study 1and 3: LapSim	· Study 1and 3: None	· Study 1: 1 month · Study 3: 5 weeks	· Study 1 and 3: None specified*	· Camera navigation · Instrument navigation · Coordination · Grasping · Lifting and grasping · Cutting · Clip applying
**Hung et al., 2012**	· 24 robotic surgery trainees	· Completed fewer than 10 robotic cases	· Vinci Si	· None	· 5 weeks	· 45 minutes	· Run bowl and cut on circumferentially inked line on bowl · Cut 2.5-cm inked line on anterior surface of bladder and water tight repair · Resect Styrofoam tumor with a clean margin of renal parenchyma
**Korndorffer et al., 2005**	· 17 surgical residents PG1-PG5	· Demographic · Video game ability	· MISTELS	· None	· 8 weeks	· 8 hours (8 weeks during 1-hour weekly sessions)	· Trainees trained on suturing.
**Larsen et al., 2009**	· 21 1st and 2^nd^ year student specializing in OB/GYN	· None	· LapSim Gyn	· None	· Unclear*	· 7 hours and 15 minutes	· Trained on “lifting and grasping” and “cutting” [AND performed salpingectomy sparing ovary · 0 and 30 degree camera manipulation, hand-eye coordination, clipping, grasping and clipping, two handed maneuvers, cutting, fulguration, and object translocation.
**Seymour et al., 2002**	16 PGY1-4 surgical residents	· Visuospatial, perceptual · Psychomotor ability tests	· MIST-VR	· Video demonstrating optimal procedure performance	· No initial assessment other than ability tests	· 1 hour	· Manipulate and diathermy task
**Stefanidis et al., 2008**	· 32 medical students 1) 6 control group 2) 13 trained group 3) 13 trained group plus environmental and more complex	· Demographic data · Simulator experience · Laparoscopic experience · NASA TLX work load	· FLS video trainer model	· Assessed on the trainer for retention before being assessed for transfer on Porcine Model	· Retention and transfer tests conducted on same day. · Average time between baseline and completion of training was 8.4 days	· For group II average training time was 239 minutes · For group III average training time was 329 minutes	· Group II a. Trained to proficiency in lap suturing on an FLS video trainer model. · Group III · Trained until proficiency · Perform the task in a constrained space · Had to listen to OR noise through headphones · Had to practice with shorter suture · Had to start with a dropped needle whose tip was facing away from the FLS model. · These four conditions were introduced gradually.
**Stefanidis et al., 2007**	· 15 novices	· Demographics · Experience with surgery and simulators	· Simulator	· None	· Not specified (approximately from 4-5)*	· Average training was 4.7 hours (1.2 SD) · 41 reps (10 SD) · Lasted 6 days (4 SD)	· Laparoscopic suturing was assessed
**Sroka et al., 2010**	· 16 surgical residents (PGY 1-3) with no prior Fundamentals of Laparoscopic surgery training 1) 8 trained 2) 8 control	· None	· MISTELS · Box Trainer	· None	· Mean time between pre and post training evaluations was 145 days.	· Average time training on the simulator was 450 minutes.	· Peg transfer · Circle cut · Placement of a ligating loop · Simple suture tied with extra and intracorporeal techniques
**Van Sickle et al. 2008**	· 22 senior surgical residents (PGY3-6) 1) 11 control group 2) 11 trained group (Simulation and box trainer)	· Demographic · Perceptual ability · Previous laparoscopic surgery experience	· MIST-VR · Box trainer	· None	· Not specified*	· Not specified*	· Suturing on the VR trainer and box trainer · Knot tying on the box trainer
**Verdaasdonk et al., 2008**	· 19 surgical trainees (1^st^ and 2^nd^ year) 1) 10 control group 2) 9 training group	· None	· SIMENDO VR simulator	· None	· 1 week	· Not specified*	· Double surgical knot tying
**Zendejas et al., 2011**	· 50 PGY 1-5 1) 26 trained group 2) 24 control group	· Demographics · Video game ability	· Guildford MATTU TEP task trainer	· None	· Approximately 10 days	· Unclear	· Trainees reduced the hernia sacs of right-sided indirect and femoral hernias and to position and tack a piece of 3.5 inches x 5 inches polypropylene mesh over the myopectineal orifice covering all potential right sided hernia defects.

* Note: Indicates articles that are unclear or do not supply an explanation of information.

**Table 3:** Published reference, context of final assessment, source of assessment, skills assessed post-training, and results from studies

Citation	Contextual setting for final assessment	Source of final assessment ratings	Skills assessed post training	Results from research studies
**Aggarwal et al., 2007**	Porcine Model (pre on box trainer)	2 observers (OSATS global rating and a motion tracking device)	· Change in operative performance: 1) time taken 2) total path length 3) total number of movements · The OSATS global rating scale.	· Trained group performed significantly better on time (p=.038), total path length (p=.001), total number of movements (p=.009) and overall rating scores (p=.001). · Trained group demonstrated dexterity scores equivalent to expert levels.
**Ahlberg et al., 2007**	Porcine Model			· The performance with MIST-VR correlated with surgery skills. · MIST-VR did not improve surgical skills · MIST-VR did predict surgical outcomes.
**Ahlberg et al., 2002**	Patients in OR (pre on a simulator)	2 observers (reliability greater .98)	· Exposure errors, clipping and tissue division errors, and dissection errors · Performance was broken up into phases: 1) exposure of the cystic duct and artery 2) clip placement followed by division of the cystic duct and artery; and 3) gallbladder excision. · Total time, path length, angular path, tissue damage, and max damage	· Intervention group made significantly fewer errors. · The trained group made significantly fewer objectively assessed, intraoperative errors during the exposure portion of the procedure (p<.04), clipping and tissue division (p<.008), and dissection (p<.03). · The control group made 3 times as many errors and used 58% longer surgical time
**Andreatta et al., 2006**	Porcine Model	2 surgeons (.99 reliability)	· 30-degree Camera navigation: 1) Time 2) Accuracy 3) Efficiency of motion 4) Instrumentation use · Eye-hand coordination: two handed transfer of ski needle: 1) Time 2) Accuracy 3) Efficiency of motion 4) Instrument handling · Eye-hand coordination: 0-degree camera navigation and one-handed object transfer: 1) Time 2) Accuracy 3) 0-degree camera navigation skills 4) perceptual ability · Safe placement of clips and application of electrocautery: 1) Clipping 2) Electrocautery performance	· Intervention group outperformed the control group in: camera navigation skills (p<.05), efficiency of motion (p<.001), optimal instrument handling (p<.001), perceptual ability (p<.001), and performance of safe electrocautery (p<.01). · Time and accuracy ratings on 30-degree navigation (p<.05), and eye-hand coordination two-handed transfer of ski needle (p<.001) was better in the trained group. · Prior training with LapMentor leads to improved resident performance of basic skills in the animate operation room.
**Banks et al., 2007**	Patients in OR (post only. Pre assess was done on simulator and then the training group performed on the simulator again before being evaluated in the OR)	Observers	· Task specific checklist: assessed 4 categories of skills: 1) preoperative skills 2) surgical technique 3) laparoscopic technique 4) laparoscopic BTL-specific skills · Global rating scale: 1) respires for tissue 2) time and motion 3) instrumental handling 4) knowledge of instruments 5) flow of operation 6) use of assistants 7) knowledge of the specific procedure · Pass/fail	· Intervention group performed significantly better than control group on all 3 surgical assessment tools (p=.002, checklist; p=.003, global score; p=.003, pass rate; p=.003, posttest) and scored significantly better on the knowledge posttest (p=.009)
**Bennet et al., 2011**	Patients in OR (post only)	Observers	· Identification of all 4 target numbers and the ability to maintain correct orientation of the camera at each target and to properly position the post at each target for a maximum total score of 12 points. · Max time was 120 seconds.	· No difference in learning between groups (p=.40).
**Gala et al., 2013**	Patients in OR	Observers	· Time · Competence levels of participants pre and post intervention · Technical skills for both groups	· Time the intervention group improved significantly higher (*p*<.01) · Intervention group was significantly higher with competence levels (*p*<.01) · The intervention group also had higher technical skills in the operating room (p<.03)
**Ganai et al., 2007**	Porcine Model (pre and post)	3 External observers (90%) and from Endo Tower simulator	· 12 structured scope navigation tasks in 3 phases: 1) Navigation within the peritoneal cavity 2) Navigation around the retracted gallbladder 3) Navigation around a suspended small intestinal loop	· Intervention group was significantly better in object visualization (p<.05), scope orientation (p<.05), and horizon errors (p<.05)
**Grantcharov et al., 2004**	Patients in OR	2 Senior surgeons rated 1 surgery (cohen’s kappa .71)	· Economy of movement: 1) Unnecessary movements 2) Confidence of movements · Errors: 1) Respect for tissue 2) Precision of operative technique	· Intervention group showed greater improvement in error (p=.003) and economy of movement (p=.003). · Intervention group was significantly faster than the control group when performing cholecystectomy (p=.021).
**Hogleet al., 2009**	Study 1: OR Patients Study 3: Porcine Model (pre and post)	Study 1: Attending surgeon Study 3: Observer	· Study 1 and 3: GOALS rating: 1) Depth perception 2) Bimanual dexterity 3) Efficiency 4) Tissue handling and autonomy	· Study 1 and 3: No significant differences were found between groups.
**Hung et al., 2012**	Porcine Model	3 expert robotic surgeons blinded	· GOALS: 1) Depth perception 2) Bimanual dexterity 3) Efficiency 4) Tissue handling 5) Participant autonomy to accomplish task	· Groups 1 and 2 were comparable in pre-study surgical experience and had similar baseline scores on simulator and tissue exercises (p > 0.05). · Overall baseline simulator performance significantly correlated with baseline and final tissue performance (p <0.0001) · Simulator training significantly improved tissue performance on key metrics for group 1 subjects with lower baseline tissue scores than their group 2 counterparts (p < 0.05) · Group 1 tended to outperform group 2 on final tissue performance, although the difference was not significant.
**Korndorffer, et al., 2007**	Porcine Model (pre and post)	Observers	· Time · Accuracy errors · Knot security	· The training group and the control group demonstrated significant improvement in completion time, and overall score. · The training group also demonstrated significant improvement in accuracy errors. · The trained group performed significantly better in completion time and overall score when comparing posttest scores to the control group. · Intervention group performed significantly better than control group
**Larsen et al., 2009**	Patients in OR (post only, pre was on a VR Simulator)	Observers	· Primary outcome measure: 1) technical performance using the objective structured assessment of laparoscopic salpingectomy 2) 5-items general rating scale and five-item task specific rating scale. · Time	· Intervention group gained experience equivalent to 20-50 procedures. · The median score on general and task specific scale reached 33 points for the trained group and 23 in the control group (p<.001). · The median score for time was 12 minutes for the trained group and 24 minutes for the control group (p<.001).
**Seymour et al., 2002**	Patients in OR (post only, pre was only ability tests)	Observers	· Operative errors 1) lack of progress 2) gallbladder injury 3) liver injury 4) incorrect plan of dissection 5) burn nontarget tissue 6) tearing tissue 7) instrument out of view 8) attending takeover	· Intervention group was faster for gallbladder dissection (29% faster), and control group was more likely to fail to make progress (Z=-2.677, p<.008) and more likely to injure the gallbladder or burn non-target tissue (5times more likely, Chi square=4.27, p<.039). · The mean number of scored errors per procedure was significantly greater in the control group than the trained group (p=-2.76, p<.006).
**Stefanidis et al., 2008**	Porcine Model (pre and post)	Objective scores based on time and errors using a published formula	· Time · Errors	· Intervention group performed substantially better than control group (p<.001). · Proficiency-based simulator training results in improved operative performance.
**Stefanidis et al., 2007**	Porcine Model (pre and post) A posttest was taken right after training was done, and then a retention test was taken after 5 months	Observers	· Errors · Time	· Intervention group outperformed control group (p<.001). · Proficiency-based simulator training results in durable improvement in operative skill of trainees even in the absence of practice for 5 months.
**Sroka et al., 2010**	MISTELS and Box Trainer on Patients in the OR	Attending surgeon or external evaluator	· FLS ratings and GOALS ratings: 1) Depth perception 2) Bimanual dexterity 3) Tissue handling 4) Efficiency 5) Autonomy	**FLS scores** · Scores increased and SD decreased in the trained group as compared to the non-trained group (p=.004). At baseline no participant had reached the required FLS scores. · Post training 100% of the trained group reached required scores and 37.5% of the non-trained reached required passing scores. **GOALS scores** · The trained group improved significantly and clinically by a mean of 6.1 +/- 1.3 (p = .0005 vs. control, and p <.0001 vs. baseline)Gender was examined as a covariate and results remained the same, trained group scores were significantly better than the control group (p=.001)Of the 5 individual domains evaluated by the GOALS rating structure greater improvements were shown in the specific domains than the generic domains for the trained group (bimanual dexterity, p=.04; depth perception p=.08; tissue handling p=.04)
**Van Sickle et al., 2008**	Patients in OR (post only)	2 surgeons (agreement > .80)	· Suturing operative errors	· Intervention group performed significantly faster (p<.003), made fewer errors (p<.01), and fewer excess needle manipulation (p<.05).
**Verdaasdonk et al., 2008**	Porcine Model (post only)	2 Expert laparoscopic surgeons	· Observer rated error assessments · Global ratings of knot tying economy of movements · Error assessments	· Intervention group tied knots faster (30%, p=.034) and made fewer errors (33%) as compared to control group. · Experimental group dropped the needle fewer times and made less frequent unnecessary contact with the tip of the needle against the tissue tan the control group (p<.05). · No significant differences in the scores assigned to the groups by the two experts (economy of movement p=.114; error assessment p=.148).
**Zendejas et al., 2011**	OR (pre and post)	Observers and medical records	· Operative performance by using a global rating using: 1) GOALS 2) operating time 3) proportion of procedure performed by the trainee 4) need for overnight stay 5) recurrence of inguinal hernia and chronic groin pain and complications.	· The trained group were on average 6.5 minutes faster than the control group (p<.0001). · Resident participation was also different between the groups with the trained group performing more of the procedure than the control group (88% vs. 73%). · After correcting time to account for varying participation rates, the trained group performed the procedure 13.1 minutes faster. · The trained group had higher performance scores than the trained group (p=.001). · Intraoperative and postoperative complicates and overnight stay were less likely in the trained group than the control group p<.05. · When follow ups with patients were conducted the number of patients who experienced a hernia recurrence or were evaluated for groin pain at least 3 month post repair there was no difference between the groups.

Note: * Indicates articles that are unclear or do not supply an explanation of information.

### 3.1 Performance Time (n = 13 Studies) (Bennett et al., 2011; Andreatta et al., 2006; Aggarwal et al., 2007; Ahlberg et al., 2007; Gala et al., 2013; Larsen et al., 2009; Grantcharov et al., 2004; Clevin, & Grantcharov, 2008; Hiemstra et al., 2011; Ganai et al., 2007; Stefanidis et al., 2008; Stefanidis et al., 2007)

Performance time was reported as the amount of time taken to perform the laparoscopic procedure of interest at the posttest evaluation. Of the 13 (62%) studies that assessed whether the training intervention resulted in the improvement of performance time, thirteen studies reported statistically significant improvement. For example, in one study researchers reported that the control group took 58% longer to perform the surgery (Ahlberg et al., 2007) and in another study investigators reported that the control group, on average, performed the surgery twice as long as the intervention group (24 minutes as compared to 12 minutes, *P* < .001) (Van Sickle et al., 2008). In yet another study the intervention group was 29% faster in dissecting the gallbladder during a cholecystectomy than the control group (Van Sickle et al., 2008). On the other hand, two studies (Bennett et al., 2011; Gala et al., 2013) reported no significant changes in time between the intervention and control groups when performance time was measured.

### 3.2 Global Ratings (n =7 studies) (Aggarwal et al., 2007; Verdaasdonk et al., 2008; Hogle et al., 2009; Sroka et al., 2010; Seymour et al., 2002; Zendejas et al., 2011; Grantcharov et al., 2004)

Global assessments were conducted using the Objective Structured Assessment of Technical Skill (Lucas, Tuncel, Bensalah, Zeltser, Jenkins, Pearle, & Cadeddu, 2008) (OSATS) rating scale, The OSATS evaluation tool evaluates participants on respect for tissue handling, time and motion, instrument handling, knowledge of instruments, flow of operation, use of assistant, and knowledge of procedure. GOALS rating scale (Watterson, Beiko, Kuan, & Denstedt, 2002) measures performance in 5 domains; three of the domains are specific to laparoscopic surgery (e.g., depth perception, bimanual dexterity and tissue handling) and 2 of the domains are generic (e.g., efficiency and autonomy). The standard Fundamentals of Laparoscopic Surgery (FLS) metrics (Larsen et al., 2009). FLS are the basic psychomotor skills necessary prior to learning how to perform and develop a laparoscopic surgical case. A different study reported that global assessment scores increased and their standard deviation decreased in the intervention group as compared to the non-trained group (*P* =.004) (Hogle, Chang, Strong, Welcome, Sinaan, Bailey, & Fowler, 2009). Moreover, in the same study 100% of intervention participants reached the passing score level where as only 37.5% of the control group. Investigators did not find any statistical significance between the two groups; however, the participants with low baseline performance increased their scores significantly after simulation training (Hung et al., 2012).

### 3.3 Suturing, Cutting and Cautery Skills (n = 3 Studies) (Andreatta et al., 2006; Ahlberg et al., 2002; Van Sickle et al., 2008)

Three (14%) of the 21 studies reported significant improvement on suturing, cutting, and cautery skills in the trained group as compared to the control group. Investigators reported that the trained participants outperformed the control participants in the performance of safe electrocautery (*P* < .01) (Andreatta et al., 2006).

Errors (*n* = 7 Studies) (Ahlberg et al., 2007; Verdaasdonk et al., 2008; Clevin, & Grantcharov, 2008; Hiemstra et al., 2011; Korndorffer Jr et al., 2005; Stefanidis, Acker,& Heniford, 2008; Stefanidis et al., 2007)

Seven (33%) of the studies assessed whether simulation-based training resulted in a decrease in errors. Errors were reported as clipping errors, dissection errors, tissue damage, incorrect plane for dissection, lack of progress, and instrument out of view. All seven-research articles reported statistical findings that the intervention decreased the amount of errors that occurred. For example, investigators that the intervention group made significantly fewer errors related to tissue division (*P*=.008) and dissection (*P*=.03) with the control group producing three times as many errors (Ahlberg et al., 2007).

*3.4 Economy of Movement* (*n* = 8 Studies) (Bennett et al., 2011; Andreatta et al., 2006; Aggarwal et al., 2007; Ahlberg et al., 2002; Gala et al., 2013; Hogle et al., 2009; Zendejas et al., 2011; Torkington et al., 2001)

Eight of the studies assessed if simulation-based training resulted in an increase in the economy of movement. Economy of movement was reported as camera navigation, efficiency of instrument, total path length, number of movements, navigation, and bimanual dexterity. The eight studies (38%) reported statistical findings that the intervention increased the economy of movement. More specifically, training was significantly related to path length (*P*<.001) and total number of movements (*P* =.009) (Aggarwal et al., 2007). In contrast, investigators found no difference in economy of movement between the control and intervention groups (*P* =.40) (Bennett et al., 2011). In two different studies, researchers found that the control groups did not show significant differences compared to the intervention group as related to economy of movement (Bennett et al., 2011; Hogle et al., 2009).

## 4. Discussion

This review of laparoscopic literature and translation of skills summarizes the evidence for the simulation-based training studies and supports skill transfer in a safe and effective way for novice surgeons to learn to perform procedures on patients in the OR (Table 3). Those responsible for teaching and assessing surgical performance should consider implications of these findings in three major areas: (1) education for competence or improved skills practiced in a controlled setting, (2) translation of new knowledge into performance outside the simulated setting, and (3) safety for patients.

## 5. Education

Laparoscopic surgery curricula may be modified or supplemented with the implementation of simulation-based training. Simulation can lead to improved assessment, improved training, error reduction, and the development of technical skills in laparoscopic surgery necessary to operate on real patients (Van Sickle et al., 2008). Residents in the intervention group made fewer errors and were less likely to injure the gallbladder or to burn non-target tissue on real patients (Van Sickle et al., 2008). Simulation-based training allows for repeated practice of standardized tasks under reproducible conditions and enables the use of objective measures for assessment purposes (Sroka, Feldman, Vassiliou, Kaneva, Fayez, & Fried, 2010) and student feedback. A simulation-based training curriculum has the potential to shorten the learning time for laparoscopic procedures compared to traditional teaching methods in laparoscopic surgery (Seymour et al., 2002).

Surgical residents who received simulation-based training curriculum significantly outperformed surgical residents who received the standard curriculum on knot tying (Zendejas et al., 2011). Additionally, surgical residents who received simulation-based training performed the suturing task faster, made fewer errors, and were more efficient in handling the suture (Zendejas et al., 2011). Overall, participants who received simulation based skills training demonstrated faster attainment of those skills than their peers from the control group in a high stakes environment (Grantcharov et al., 2004). Training curriculum related to laparoscopic surgery skills allows for more learning opportunities for novice surgeons to practice with simulation-based training prior to entering OR environment; thus, allowing for the potential of skills translating into the OR.

Finally, the studies in this review show that simulation-based training should be incorporated into surgical curricula specifically targeting novice learners. Presently, simulation-based training programs are generally offered as a supplement to traditional surgical training and are voluntary (Graber, Wyatt, Kasparek, & Xu, 2005). Currently, there is not a standard or universal specific surgical curriculum in place in surgical educational programs; however, there has been a recent change, FES (Fundamentals of Endoscopic Surgery) was approved in March, 2014 as an additional requirement for residents graduating in 2018 and after this is a simulation-based training program.

Further research is needed to determine the best longitudinal curriculum for basic and advanced skills acquisition and transfer to the OR environment. Simulation-based training will allow for the novice to learn the psychomotor skills and spatial judgments necessary for laparoscopic surgical skills allowing them to focus more on learning operative strategies and handling intraoperative complications while in the OR (Torkington, Smith, Rees, & Darzi, 2001). Training in proficiency-based skills should be incorporated into a comprehensive surgical training and assessment curriculum for residents prior to operating on real patients (Banks, Chudnoff, Karmin, Wang, & Pardanani, 2007). The pressure to make surgical training more efficient and safer for patients is substantial, and simulation-based training has the potential to improve surgical curricula (Clevin & Grantcharov, 2008).

## 6. Translation

Translational impact was achieved in the OR with live patients when simulation-based training was used for the educational intervention. Researchers found that training in a simulated environment led to improved surgical performance on either animals or humans (Ahlberg et al., 2007; Verdaasdonk et al., 2008; Ahlberg et al., 2002; Van Sickle et al., 2008; Seymour et al., 2002; Zendejas et al., 2011; Banks et al., 2007; Clevin, & Grantcharov, 2008; Crochet et al., 2011). Simulation-based training, influences the translation of laparoscopic surgery skills to the OR. As a result of these findings, simulation-based training has the potential to provide the foundational skills necessary for future surgeons to learn in a controlled environment and translate those acquired skills to the OR. With increases in technology and the need for a standard surgical curricula there is potential with simulation as an educational tool to further the translation of laparoscopic surgical skills into the OR. More specifically, typical skills that translate into the OR are suturing, camera navigation, and the manipulation of equipment.

## 7. Patient Safety

Simulation-based training has the potential to lead to an increase in patient safety. Residents who trained with simulation had fewer errors than control groups (Van Sickle et al., 2008; Hiemstra et al., 2011) while in the OR. Participants in the intervention group had fewer incidents of the supervising surgeon taking over the procedure. These types of events can significantly affect clinical outcomes because they represent potential errors in technique compromising patient safety (Ahlberg et al., 2002).

Using simulation for training surgical skills can benefit the larger goal of improved patient safety in several ways. With simulation, learners can repeat a procedure or even a specific element of a procedure until competency is demonstrated. Novice surgeons enter the OR more apt to produce favorable patient outcomes and are better prepared to participate in surgical cases with live patients in the OR if they previously trained on a simulator. Simulation can also provide more opportunities for remedial training to reduce skill decay (Sroka et al., 2010). Laparoscopic surgical simulators provide opportunities to train other concepts central to patient safety. For example, teamwork skills can be trained through surgeons interacting with camera navigators or nurses in a simulated OR. Simulating laparoscopic surgical equipment and interfaces can even be used to introduce, test, and train new equipment or protocols before they are implemented in the OR, leading to identification of potential latent threats to safety and avoidance of medical errors due to poor human systems integrations.

## 8. Limitations

As with any literature review, our review and results are limited by the data provided in the original studies. Our findings are limited by the lack of descriptions of the data collection process and interventions of the included studies. In particular, it was difficult to discern many of the potential covariates that were used in the data analyses as well as the timing between pre- and post-tests once the interventions were implemented. Moreover, a majority of the studies that reported statistical results reported the results using p-values. The lack of effect size reporting contributes to the difficulty in truly understanding the magnitude of the effect of these interventions on the acquisition of surgical skills.

Another limitation to this study is this was only one database was used to identify all literature, data, or studies related to a specific topic. Therefore, potentially, excluding conference presentations, other online search engines, and contacting colleagues within the field to identify any potential missing studies that may not have been included. Furthermore, not all surgical journals were hand searched, just those identified by one author as to be key surgery journals within the field.

The scope of our review is both a strength and limitation. Restricting our scope to only randomized control trials increased the stability of the findings reported in the original studies. However, it is not possible to draw firm conclusions about the effectiveness of the different types of simulation based on our findings as many of the RCTs did not conduct comparative analyses between varying types of simulations. Nonetheless, we argue that our review does provide useful insight into the literature that examines the effectiveness of simulation based laparoscopic training interventions. The need for more robust comparisons of these training interventions is needed to be able to provide an unequivocal conclusion to the impact on surgical skills.

## 9. Conclusion

Simulation-based training can lead to demonstrable benefits of surgical skills in the OR. These benefits include decreased procedural errors as well as other effects on overall patient safety. This review suggests that simulation-based training is an effective way to teach laparoscopic surgery skills, increase translation of laparoscopic surgical skills to the OR and increase patient safety. However, more research should be conducted to determine if and how simulation can become apart of the surgical curriculum.

## References

[ref1] Aggarwal R, Ward J, Balasundaram I, Sains P, Athanasiou T, Darzi A (2007). Proving the effectiveness of virtual reality simulation for training in laparoscopic surgery. Annals of surgery.

[ref2] Ahlberg G, Enochsson L, Gallagher A. G, Hedman L, Hogman C, McClusky D. A, Arvidsson D (2007). Proficiency-based virtual reality training significantly reduces the error rate for residents during their first 10 laparoscopic cholecystectomies. The American journal of surgery.

[ref3] Ahlberg G, Heikkinen T, Iselius L, Leijonmarck C. E, Rutqvist J, Arvidsson D (2002). Does training in a virtual reality simulator improve surgical performance?. Surgical Endoscopy and Other Interventional Techniques.

[ref4] Andreatta P. B, Woodrum D. T, Birkmeyer J. D, Yellamanchilli R. K, Doherty G. M, Gauger P. G, Minter R. M (2006). Laparoscopic skills are improved with LapMentor™ training: results of a randomized, double-blinded study. Annals of surgery.

[ref5] Banks E. H, Chudnoff S, Karmin I, Wang C, Pardanani S (2007). Does a surgical simulator improve resident operative performance of laparoscopic tubal ligation?. American Journal of obstetrics and gynecology.

[ref6] Bennett A, Birch D. W, Menzes C, Vizhul A, Karmali S (2011). Assessment of medical student laparoscopic camera skills and the impact of formal camera training. The American journal of surgery.

[ref7] Clevin L, Grantcharov T. P (2008). Does box model training improve surgical dexterity and economy of movement during virtual reality laparoscopy?A randomized trial. Obstetrical & Gynecological Survey.

[ref8] Cook D. A, Hatala R, Brydges R, Zendejas B, Szostek J. H, Wang A. T, Hamstra S. J (2011). Technology-enhanced simulation for health professions education: a systematic review and meta-analysis. Jama.

[ref9] Crochet P, Aggarwal R, Dubb S. S, Ziprin P, Rajaretnam N, Grantcharov T, Darzi A (2011). Deliberate practice on a virtual reality laparoscopic simulator enhances the quality of surgical technical skills. Annals of surgery.

[ref10] Ehdaie B, Tracy C, Reynolds C, Cung B, Thomas K, Floyd T, Schenkman N (2011). Evaluation of laparoscopic curricula in American urology residency training. Journal of Endourology.

[ref11] Gala R, Orejuela F, Gerten K, Lockrow E, Kilpatrick C, Chohan L, Schaffer J (2013). Effect of validated skills simulation on operating room performance in obstetrics and gynecology residents: a randomized controlled trial. Obstetrics & Gynecology.

[ref12] Ganai S, Donroe J. A, St Louis M. R, Lewis G. M, Seymour N. E (2007). Virtual-reality training improves angled telescope skills in novice laparoscopists. The American journal of surgery.

[ref13] Graber M. A, Wyatt C, Kasparek L, Xu Y (2005). Does simulator training for medical students change patient opinions and attitudes toward medical student procedures in the emergency department?. Academic emergency medicine.

[ref14] Grantcharov T. P, Kristiansen V. B, Bendix J, Bardram L, Rosenberg J, Funch-Jensen P (2004). Randomized clinical trial of virtual reality simulation for laparoscopic skills training. British Journal of Surgery.

[ref15] Gurusamy K, Aggarwal R, Palanivelu L, Davidson B. R (2008). Systematic review of randomized controlled trials on the effectiveness of virtual reality training for laparoscopic surgery. British Journal of Surgery.

[ref16] Hiemstra E, Terveer E. M, Chmarra M. K, Dankelman J, Jansen F. W (2011). Virtual reality in laparoscopic skills training: Is haptic feedback replaceable?. Minimally Invasive Therapy & Allied Technologies.

[ref17] Higgins J. P. T (2012). Green S. Cochrane handbook for systematic reviews of interventions Version 5.1. 0 [updated March 2011]. The Cochrane Collaboration, 2011. www.cochrane-handbook.Org.

[ref18] Hogle N. J, Chang L, Strong V. E. M, Welcome A. O. U, Sinaan M, Bailey R, Fowler D. L (2009). Validation of laparoscopic surgical skills training outside the operating room: a long road. Surgical endoscopy.

[ref19] Hung A. J, Patil M. B, Zehnder P, Cai J, Ng C. K, Aron M, Desai M. M (2012). Concurrent and predictive validation of a novel robotic surgery simulator: a prospective, randomized study. The Journal of urology.

[ref20] Hyltander A, Liljegren E, Rhodin P. H, Lönroth H (2002). The transfer of basic skills learned in a laparoscopic simulator to the operating room. Surgical Endoscopy and Other Interventional Techniques.

[ref21] Johnson M. D, Walsh R. M (2009). Current therapies to shorten postoperative ileus. Cleveland Clinic journal of medicine.

[ref22] Jordan J. A, Gallagher A. G, McGuigan J, McGlade K, McClure N (2000). A comparison between randomly alternating imaging, normal laparoscopic imaging, and virtual reality training in laparoscopic psychomotor skill acquisition. The American journal of surgery.

[ref23] Korndorffer J. R, Dunne J. B, Sierra R, Stefanidis D, Touchard C. L, Scott D. J (2005). Simulator training for laparoscopic suturing using performance goals translates to the operating room. Journal of the American College of Surgeons.

[ref24] Larsen C. R, Soerensen J. L, Grantcharov T. P, Dalsgaard T, Schouenborg L, Ottosen C, Ottesen B. S (2009). Effect of virtual reality training on laparoscopic surgery: randomised controlled trial. Bmj.

[ref25] Liberati A, Altman D. G, Tetzlaff J (2009). The PRISMA statement for reporting systematic reviews and meta-anlyses of studies that evaluate healthcare interventions: exploration and elaboration. Br. Med. J.

[ref26] Lucas S, Tuncel A, Bensalah K, Zeltser I, Jenkins A, Pearle M, Cadeddu J (2008). Virtual reality training improves simulated laparoscopic surgery performance in laparoscopy naive medical students. Journal of Endourology.

[ref27] McGaghie W. C, Siddall V. J, Mazmanian P. E, Myers J (2009). American College of Chest Physicians Health and Science Policy Committee. Lessons for continuing medical education from simulation research in undergraduate and graduate medical education: effectiveness of continuing medical education: American College of Chest Physicians Evidence-Based Educational Guidelines. Chest.

[ref28] Miskovic D, Wyles S. M, Ni M, Darzi A. W, Hanna G. B (2010). Systematic review on mentoring and simulation in laparoscopic colorectal surgery. Annals of surgery.

[ref29] Munz Y, Kumar B. D, Moorthy K, Bann S, Darzi A (2004). Laparoscopic virtual reality and box trainers: is one superior to the other?. Surgical Endoscopy And Other Interventional Techniques.

[ref30] Richardson W. S, Carter K. M, Fuhrman G. M, Bolton J. S, Bowen J. C (2000). Minimally Invasive Abdominal Surgery. The Ochsner Journal.

[ref31] Seropian M, Lavey R (2010). Design considerations for healthcare simulation facilities. Simulation in Healthcare.

[ref32] Seymour N. E, Gallagher A. G, Roman S. A, O’Brien M. K, Bansal V. K, Andersen D. K, Satava R. M (2002). Virtual reality training improves operating room performance: results of a randomized, double-blinded study. Annals of surgery.

[ref33] Sroka G, Feldman L. S, Vassiliou M. C, Kaneva P. A, Fayez R, Fried G. M (2010). Fundamentals of laparoscopic surgery simulator training to proficiency improves laparoscopic performance in the operating room—a randomized controlled trial. The American journal of surgery.

[ref34] Stefanidis D, Acker C, Heniford B. T (2008). Proficiency-based laparoscopic simulator training leads to improved operating room skill that is resistant to decay. Surgical innovation.

[ref35] Stefanidis D, Korndorffer J. R, Markley S, Sierra R, Heniford B. T, Scott D. J (2007). Closing the gap in operative performance between novices and experts: does harder mean better for laparoscopic simulator training?. Journal of the American College of Surgeons.

[ref36] Sturm L. P, Windsor J. A, Cosman P. H, Cregan P, Hewett P. J, Maddern G. J (2008). A systematic review of skills transfer after surgical simulation training. Annals of surgery.

[ref37] Sutherland L. M, Middleton P. F, Anthony A, Hamdorf J, Cregan P, Scott D, Maddern G. J (2006). Surgical simulation: a systematic review. Annals of surgery.

[ref38] Torkington J, Smith S. G. T, Rees B. I, Darzi A (2001). Skill transfer from virtual reality to a real laparoscopic task. Surgical endoscopy.

[ref39] Van Sickle K. R, Ritter E. M, Baghai M, Goldenberg A. E, Huang I. P, Gallagher A. G, Smith C. D (2008). Prospective, randomized, double-blind trial of curriculum-based training for intracorporeal suturing and knot tying. Journal of the American College of Surgeons.

[ref40] Verdaasdonk E. G. G, Dankelman J, Lange J. F, Stassen L. P. S (2008). Transfer validity of laparoscopic knot-tying training on a VR simulator to a realistic environment: a randomized controlled trial. Surgical endoscopy.

[ref41] Watterson J. D, Beiko D. T, Kuan J. K, Denstedt J. D (2002). A randomized prospective blinded study validating acquistion of ureteroscopy skills using a computer based virtual reality endourological simulator. The Journal of urology.

[ref42] Zendejas B, Cook D. A, Bingener J, Huebner M, Dunn W. F, Sarr M. G, Farley D. R (2011). Simulation-based mastery learning improves patient outcomes in laparoscopic inguinal hernia repair: a randomized controlled trial. Annals of surgery.

